# The Cancer Stem Cell Inhibitor Napabucasin (BBI608) Shows General Cytotoxicity in Biliary Tract Cancer Cells and Reduces Cancer Stem Cell Characteristics

**DOI:** 10.3390/cancers11030276

**Published:** 2019-02-26

**Authors:** Marlena Beyreis, Martin Gaisberger, Martin Jakab, Daniel Neureiter, Katharina Helm, Markus Ritter, Tobias Kiesslich, Christian Mayr

**Affiliations:** 1 Institute of Physiology and Pathophysiology, Paracelsus Medical University, 5020 Salzburg, Austria; marlena.beyreis@pmu.ac.at (M.B.); martin.gaisberger@pmu.ac.at (M.G.); martin.jakab@pmu.ac.at (M.J.); markus.ritter@pmu.ac.at (M.R.); tobias.kiesslich@pmu.ac.at (T.K.); 2 Ludwig Boltzmann Institute for Arthritis and Rehabilitation, Paracelsus Medical University, 5020 Salzburg, Austria; 3 Gastein Research Institute, Paracelsus Medical University, 5020 Salzburg, Austria; k.helm@salk.at; 4 Institute of Pathology, Paracelsus Medical University/Salzburger Landeskliniken (SALK), 5020 Salzburg, Austria; d.neureiter@salk.at; 5 Cancer Cluster Salzburg, 5020 Salzburg, Austria; 6 Department of Internal Medicine I, Paracelsus Medical University/Salzburger Landeskliniken (SALK), 5020 Salzburg, Austria

**Keywords:** Napabucasin, BBI608, biliary tract cancer, cancer stem cells

## Abstract

Biliary tract cancer is a devastating disease with limited therapeutic options. The involvement of cancer stem cells in biliary tract cancer is likely. Napabucasin is a previously described cancer stem cell inhibitor that is currently being used in clinical trials. However, data regarding napabucasin and biliary tract cancer are not available yet. We tested the general cytotoxic effect of napabucasin on a comprehensive biliary tract cancer in vitro model, using resazurin assay and Annexin V/7-AAD staining. The effect of napabucasin on functional cancer stem cell characteristics was analyzed via soft agar assay, aldehyde-dehydrogenase-1 assay, measurement of surface CD326 expression, and measurement of clonogenic growth. The evaluation of the effect of napabucasin on cancer stem cell protein and gene expression was performed using Western blot and reverse transcription-PCR-based human cancer stem cell array. Napabucasin showed a concentration- and cell line-dependent cytotoxic effect, and increased the apoptotic and necrotic cell fractions. Treatment with napabucasin significantly reduced the formation of tumor spheres and clonogenic growth, as well as CD326 surface expression. Expression of cancer stem cell markers were reduced following napabucasin treatment on the protein and mRNA levels. Our study provides first data regarding napabucasin as a promising substance for the treatment of biliary tract cancer.

## 1. Introduction

Biliary tract cancer (BTC) is a rare and fatal disease originating from transformed cells of the bile duct system. Anatomically, BTC can be classified into intrahepatic cholangiocarcinoma (IHC), extrahepatic cholangiocarcinoma (EHC), gallbladder cancer (GBC), and mixed hepatocellular-cholangiocarcinoma (HCC-CC) [1]. Symptoms are unspecific, and consequently, patients are often diagnosed at an advanced disease stage, significantly constraining the therapeutic options. Therefore, only palliative treatment is possible at an advanced stage, resulting in a median survival time of only about one year, using combined cisplatin and gemcitabine chemotherapy [2]. Development of new strategies in early detection and therapy, as well as the identification of new therapeutic targets, including the understanding of the underlying mechanisms of BTC development, is therefore of the utmost importance.

Cancer stem cells (CSCs) are defined as a cell population within the heterogeneous tumor mass that possess the abilities to self-renew and to differentiate into different (tumor cell) lineages. They are linked to tumor initiation, progression, resistance, and poor prognosis [3]. Cancer stem cells have been identified in most tumors and are characterized by a specific surface marker composition and several functional traits [3,4,5]. The involvement of cancer stem cells in BTC has been already described in several publications, although their exact role is not completely clear [6,7]. Chemotherapeutics may reduce the tumor mass of BTC, however, the resistance of CSC towards these treatments most probably hinders long-term success and leads to tumor regrowth, thus making CSCs an attractive therapeutic target in BTC [8]. In earlier studies, we demonstrated that the inhibition of epigenetic regulators stopped BTC cell proliferation and inhibited putative CSC subpopulations by reducing functional CSC characteristics, like sphere formation and aldehyde-dehydrogenase 1 (ALDH1)-positivity [9,10].

In 2015, Li et al. presented a newly synthesized anti-CSC drug termed napabucasin (BBI608), and demonstrated strong anti-CSC effects in vitro and in vivo in a broad range of cancer types [11]. In 2016, these results were confirmed by Zhang et al. on prostate CSCs [12]. They showed that napabucasin was able to kill prostate CSCs and to inhibit CSC properties, as well as the expression of stemness-related genes [12]. The clinical potential of the CSC inhibitor napabucasin is currently being underlined by 24 clinical trials (clinicaltrials.gov), in which napabucasin is being investigated as a potential clinical drug in several advanced cancer types. Initial results suggest that targeting CSCs by napabucasin leads to benefits in preventing secondary tumors and relapses [8]. However, while only a few studies have yet described the effect of napabucasin in tumor models in vitro, the existing studies are in accordance with the initial findings by Li et al. [11] and suggest napabucasin as a potent anti-tumorigenic and anti-CSC drug in different tumor types [8,12,13,14,15]. Considering the lack of effective therapeutic drugs, the dismal survival rate of BTC patients, and the likely involvement of CSCs in BTC development and progression, the current study conducts a first-time evaluation of napabucasin as a potential therapeutically relevant substance in BTC. For this purpose, we characterized the cytotoxic effects of napabucasin in a comprehensive BTC in vitro model, and analyzed the effect of napabucasin on functional CSC characteristics and CSC-related gene and protein expression.

We show that napabucasin has a profound cytotoxic effect in BTC cells and significantly reduces functional CSC characteristics, protein, and mRNA expression of CSC-related factors.

## 2. Results

### 2.1. Napabucasin Reduces Cell Viability in All Cell Lines in a Dose and Time Dependent Manner

**Figure 1:**
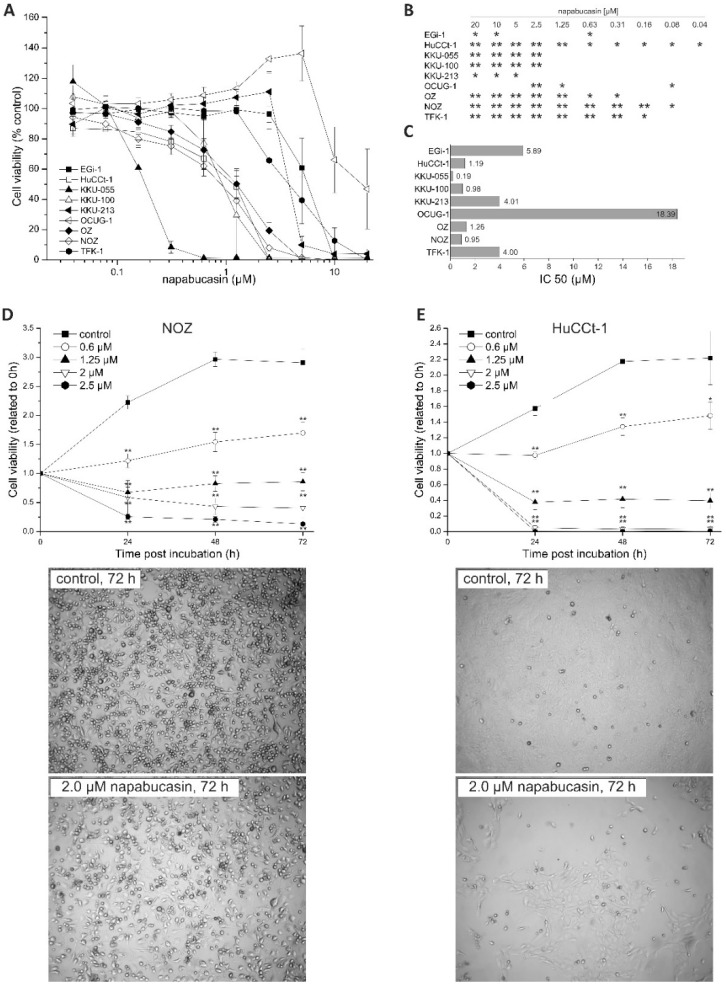
(**A**) Cytotoxic effects of napabucasin in biliary tract cancer cells. Effects of different napabucasin concentrations on cell viability of nine biliary tract cell lines after 72 h incubation period using the resazurin assay. (**B**): Statistics for Figure 1A, C: half maximal inhibitory concentration (IC_50_) values in µM of napabucasin. (**D**,**E**) Top: Time-dependent cytotoxicity of napabucasin using 0 (control), 0.6, 1.25, and 2.0 µM on NOZ (**C**) and HuCCt-1 (**D**) cells, respectively. Viability was measured after 0, 24, 48, and 72 h via the resazurin assay and related to the initial time points (0 h) for each treatment. (**D**,**E**) Bottom: Representative images of untreated and napabucasin-treated (2.0 µM) NOZ (left) and HuCCt-1 (right) cells. Pictures were taken from the center of the 96-well plates using the microplate reader. Data are presented as mean value ± standard error of the mean (SEM) related of at least three individual biological replicates * indicates significant (*p* < 0.05) and ** highly significant (*p* < 0.01) results.

We used a dilution series of napabucasin (range: 0.04-20 µM) to test the general cytotoxic effect on a panel of *n* = 9 BTC cell lines. After 72 h of incubation, cell viability was measured using the resazurin assay (metabolic activity). Napabucasin significantly reduced overall cell viability in a dose-dependent and cell line-dependent manner, ranging between 0% and 50% survival rate at high concentrations (Figure 1A,B). The cell line KKU-055 was most sensitive to napabucasin (half maximal inhibitory concentration (IC_50_): 0.19 µM), whereas the cell lines TFK-1, EGi-1, KKU-213, and OCUG-1 displayed noticeably higher IC_50_ values of up to 18 µM (Figure 1C). The remaining cell lines displayed napabucasin sensitivities, with IC_50_ values between 0.95 and 1.26 µM. For subsequent experiments, we chose the two cell lines HuCCt-1 and NOZ, as these cell lines showed high sensitivity towards napabucasin (HuCCt-1: IC_50_ 1.19 µM, NOZ: IC_50_ 0.95 µM) as well as highly reproducible and significant results over a broad range of napabucasin concentrations (Figure 1B,C). 

To get a better understanding of the cytotoxic mode of napabucasin, we next performed time-resolved analysis of cell viability. Cells were incubated with different napabucasin concentrations, and viability was measured after 0, 24, 48, and 72 h incubation time. As shown in Figure 1D,E, the time-resolved analysis of napabucasin cytotoxicity revealed concentration-dependent effects of napabucasin. In both cell lines, treatment with 0.6 µM resulted in a significant slow-down of cell growth, whereas higher concentrations (1.25, 2.0, and 2.5 µM) led to a significant reduction of viable cells below the 0 h value, indicating direct cytotoxicity (cell death). Although HuCCt-1 cells were more sensitive towards napabucasin treatment, the overall cytotoxic effect was similar between HuCCt-1 and NOZ cells. Visual assessment was performed in accordance with the resazurin measurement time points after 24, 48, and 72 h, and supported the viability assay results for both tested cell lines (Figure 1D,E and Supplementary Figure S1). 

**Figure 2:**
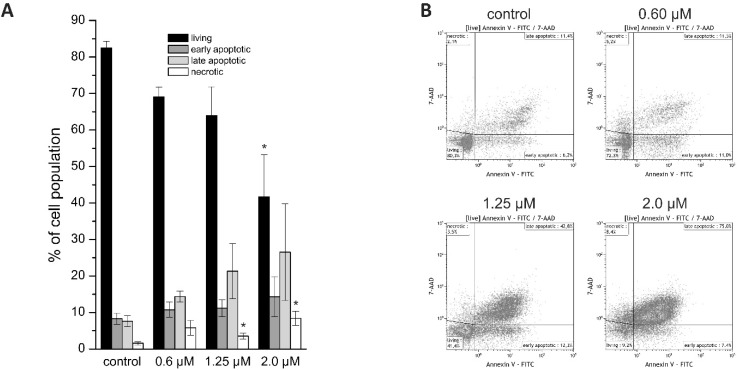
(**A**) Annexin V/7-AAD staining of HuCCt-1 cells after 24 h incubation time with 0 (control), 0.6, 1.25, or 2.0 µM napabucasin. (**B**) Exemplary Annexin V/7-AAD staining scatter plots. Data are presented as mean value ± SEM related of at least three individual biological replicates * indicates significant (*p* < 0.05) and ** highly significant (*p* < 0.01) results. FITC: fluorescein isothiocyanate.

For differentiation between living, early, and late apoptotic cells or necrotic cells, we performed Annexin V/7-AAD staining. Due to their shape and clustering following napabucasin treatment, NOZ cells were not suitable for this flow cytometry-based assay. In HuCCt1-1 cells, treatment with napabucasin led to a concentration-dependent decrease of viable cells, accompanied by a concentration-dependent increase of early and late apoptotic cells, as well as an increase of necrotic cells (significant for higher concentrations 1.25 µM and 2.0 µM) (Figure 2A,B).

### 2.2. Effects of Napabucasin on Functional Cancer Stem Cell Characteristics

**Figure 3:**
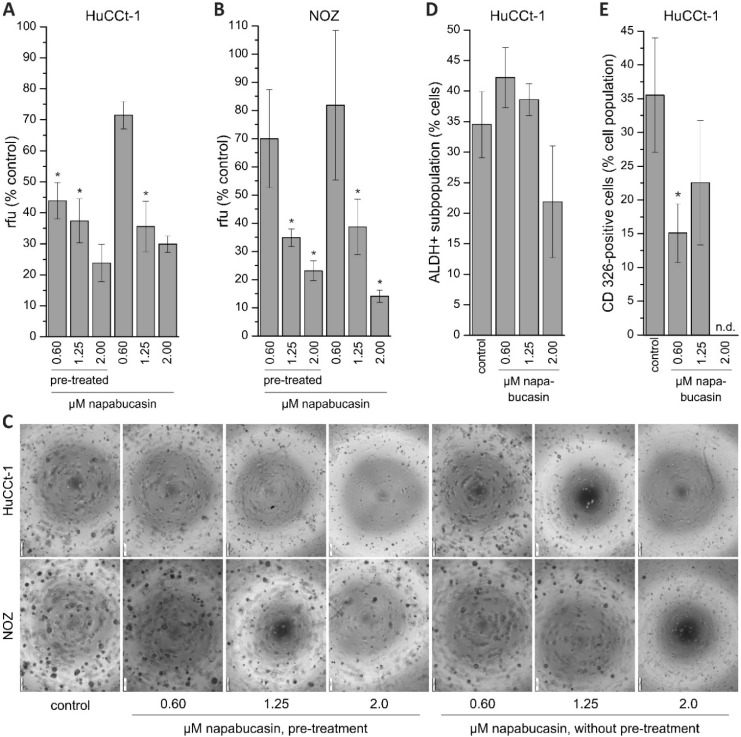
Effect of napabucasin on functional cancer stem cell characteristics. (**A**,**B**): Results of soft agar colony formation assay of HuCCt-1 and NOZ cells. Data are presented as mean value ± SEM related to untreated control cells of four individual biological replicates. Cells were either pre-treated with napabucasin or treated with napabucasin for the entire duration of the assay (eight days). (**C**) Exemplary images of the soft agar colony formation assay (scale bar at the left bottom of each image represents 200 µm. (**D**) Effect of napabucasin on the ALDH-positive subpopulation of HuCCt-1 cells (*n* = 5 individual biological replicates). (**E**) Effect of napabucasin on the surface expression of the cancer stem cell marker CD326 (*n* = 6 individual biological replicates). * indicates significant (*p* < 0.05) results. Abbreviations: ALDH: aldehyde-dehydrogenase; n.d., not determinable; rfu, relative fluorescence units.

Human stem and progenitor cells, as well as cancer stem cells, are able to survive in suspensions and create colony formations/spheres. These spheres are able to differentiate to clonally complex functional 3-dimentional structures [16]. To characterize this well-known cancer stem cell-specific function in our BTC cells, we performed soft agar colony formation assay experiments. To get a more detailed understanding of the effect of napabucasin on sphere formation, we used two approaches. In one approach, cells were pre-treated (pt) with napabucasin for 24 h, and were then seeded in the sphere formation assay in the absence of napabucasin. In the second approach, cells were not pre-treated with napabucasin, and were seeded in the sphere formation assay with media containing napabucasin. For both approaches, sphere formation was quantified after eight days. As seen in Figure 3A, the ability of HuCCt-1 to form spheres was significantly reduced in both approaches. Interestingly, pre-treatment with 0.6 µM napabucasin reduced the amount and size of colonies even more strongly when compared to non-pre-treated cells incubated with 0.6 µM napabucasin. Similarly, napabucasin reduced the sphere formation of NOZ cells (Figure 3B). In Figure 3C, representative pictures of sphere formation after 8 days for both approaches and control cells are shown for HuCCt-1 and NOZ cells. We next measured the fraction of aldehyde-dehydrogenase 1 (ALDH1) positive cells in BTC cells, with and without napabucasin treatment. ALDH1 is a detoxifying enzyme that is known to be important for maintenance of CSC status [16]. Again, NOZ cells showed strong clustering after napabucasin treatment, and were therefore not suitable for flow cytometry. About one-third of the HuCCt-1 cells were ALDH-positive without napabucasin treatment (Figure 3D). Treatment with 0.6 µM and 1.25 µM napabucasin did not diminish the ALDH-positive subpopulation, and resulted in a slight increase of ALDH-positive cells. However, treatment with 2.0 µM napabucasin reduced the number of ALDH-positive cells by one-third. CD326 is a promising candidate marker for CSC subpopulations in BTC models [6]. We therefore measured the effect of napabucasin treatment on the portion of CD326-positive cells. Treatment of HuCCt-1 cells with 0.6 µM and 1.25 µM napabucasin resulted in a significant reduction of the CD326-positive subpopulation after 24 h of incubation (Figure 3E). Due to the already high cytotoxic effect of napabucasin at 2.0 µM, measurement of CD326 surface expression was not possible at this concentration. 

### 2.3. Napabucasin Abolishes Clonogenic Growth In Vitro

**Figure 4:**
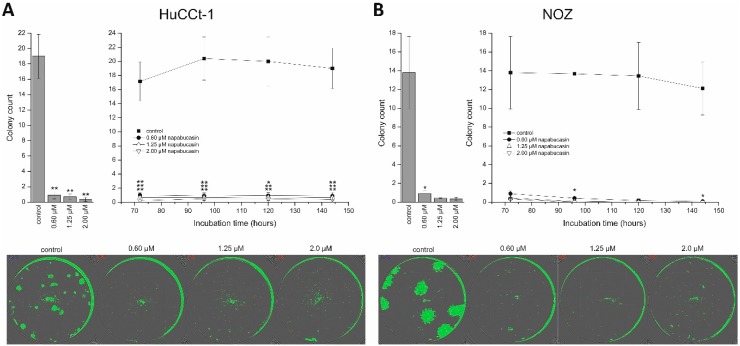
Napabucasin abolishes clonogenic growth in biliary tract cancer cells. (**A**) Quantification of clonogenic growth of HuCCt-1 cells after 144 h napabucasin incubation (left) and as a time-resolved analysis of the same wells (right). Exemplary confluence images after 144 h of napabucasin are shown in the lower part. (**B**) Quantification of clonogenic growth of NOZ cells after 144 h napabucasin incubation (left) and as a time-resolved analysis of the same wells (right). Exemplary confluence images after 144 h of napabucasin are shown in the lower part. Data are presented as mean value ± SEM related to untreated control cells of at least three individual biological replicates. * indicates significant (*p* < 0.05) and ** highly significant (*p* < 0.01) results.

Clonogenic assay is a widely used method for studying the ability of cells to grow at low seeding densities, which is interpreted as a characteristic of cells with (cancer) stem cell character [17]. As described in our previous work, we performed the clonogenic assay in 96-well microplate using the confluence function of a multimode reader [18]. By doing this, we were able to perform non-endpoint measurement of clonogenic growth, thereby gathering detailed information about the effect of napabucasin on this CSC trait. HUCCt-1 cells were grown overnight, incubated with 0.60, 1.25, or 2.00 µM napabucasin, and clonogenic growth was monitored after 72, 96, 120, and 144 h, respectively. As shown in Figure 4A, after 144 h incubation, clonogenic growth was significantly suppressed by napabucasin to <10% of the number of colonies in the control sample. Time-resolved measurement of clonogenic growth revealed that this effect was already present after 72 h (Figure 4A, right). Although the growth pattern of the clones was very different between HuCCt-1 and NOZ cells (Figure 4A,B), similar results were obtained for NOZ cells. Even the lowest napabucasin concentration (0.60 µM) was able to completely abolish clonogenic growth after 72 h incubation time (Figure 4B). 

### 2.4. Napabucasin Reduces Protein Levels of Established Cancer Stem Cells Markers

**Figure 5:**
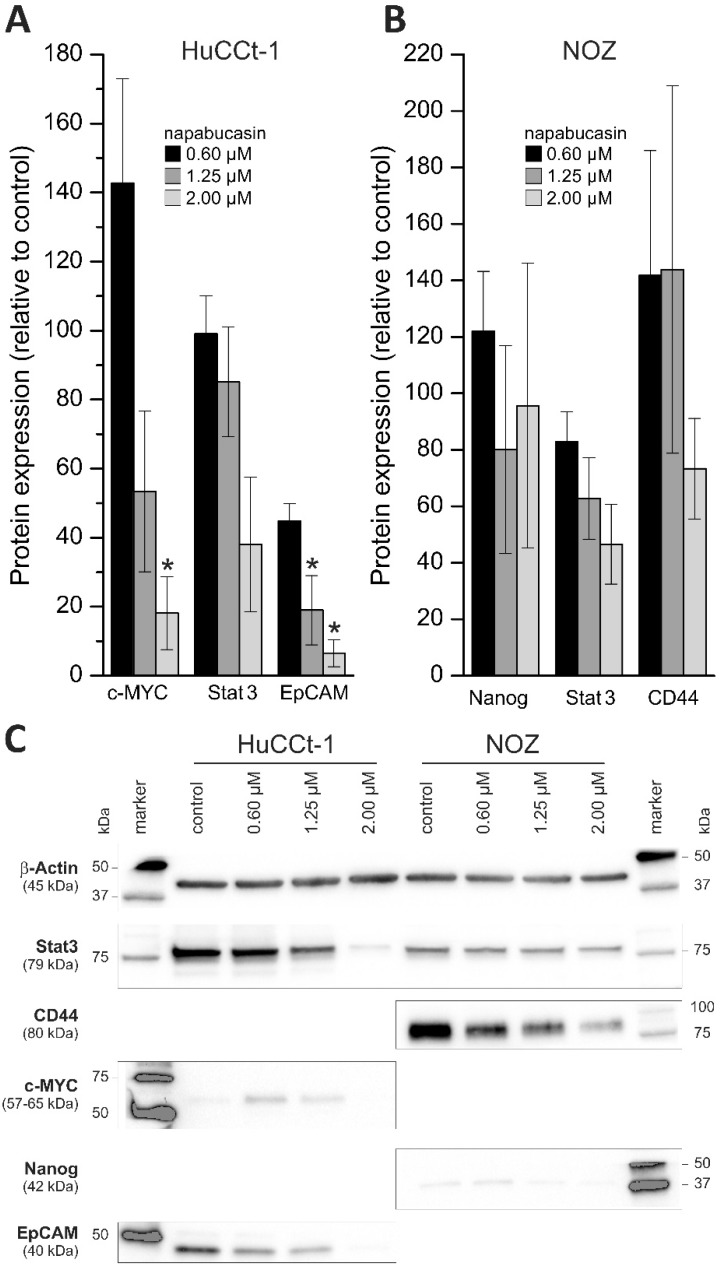
Effect of napabucasin on protein levels of established cancer stem cell markers. (**A**,**B**) Changes in protein expression of established cancer stem cell markers after 24 h treatment with 0.6, 1.25, or 2.0 µM napabucasin related to untreated control cells. (**C**) Representative Western blot images of HuCCt-1 and NOZ cells (cropped). Data are presented as mean value ± SEM related to untreated control cells of at least three individual biological replicates. * indicates significant (*p* < 0.05) results.

To get a general idea whether napabucasin affects protein levels of CSC markers, we performed Western blot analysis using a panel of established CSC markers (c-Myc, Stat3, EpCAM, Nanog, CD44, SOX2, ABCG2, p-Stat3, OCT-4A), and measured protein levels with or without napabucasin treatment in HuCCt-1 and NOZ cells. Of these markers, in HuCCt-1 cells, only c-Myc, Stat3 and EpCAM were detectable. Treatment of HuCCt-1 cells with napabucasin resulted in down-regulation of all three CSC markers after 24 h (Figure 5A,C). It should be noted that c-Myc was only down-regulated when cells were treated with 1.25 µM and 2.00 µM napabucasin. In NOZ cells, Nanog, Stat3 and CD44 were detectable and similar to HuCCt-1 cells, these markers showed a concentration-dependent down-regulation after napabucasin treatment (Figure 5B,C and Supplementary Table S1).

### 2.5. Array-Based Effects of Napabucasin on mRNA Levels of Cancer Stem Cell Markers

**Figure 6:**
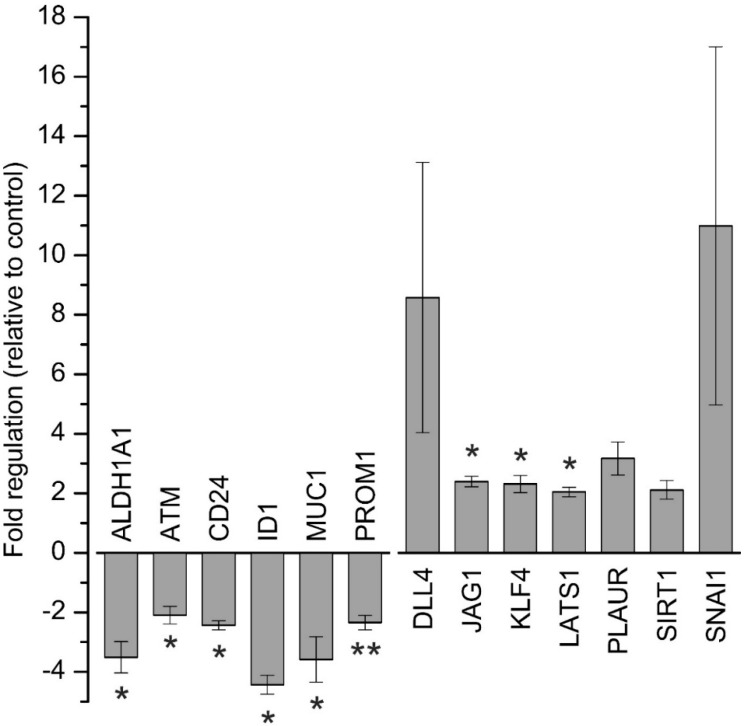
Array-based gene expression analysis of cancer stem-cell related genes after napabucasin treatment. Gene expression analysis of human cancer stem cell-related genes after 24 h of 1.25 µM napabucasin treatment using a RT^2^ Profiler Array. Data were normalized to five housekeeping genes (*β–ACTIN*, *B2M*, *GAPDH*, *HPRT1*, and *RPLP0*) according to the manufacturer’s suggestion (Qiagen Data Analysis Center) and related to untreated controls. Data are presented as mean value ± SEM related to untreated control cells of five individual biological replicates. For full gene names and RefSeq numbers see Supplementary Table S2. * indicates significant (*p* < 0.05) results.

Based on the observed downregulation of CSC-related proteins, we next aimed to investigate the influence of napabucasin on CSC factors and signalling on a more comprehensive scale. Therefore, we used the RT^2^ Profiler PCR Array for human CSC (Qiagen, Hilden, Germany), covering 84 CSC-related genes to get a broader understanding of the effect of napabucasin on CSC-related genes. To allow inclusion of sufficient replicates and consequently proper interpretation of the results, these profiling experiments were performed with one cell line (HuCCt-1) and one napabucasin treatment condition (1.25 µM; incubation time 24 h). We considered genes with a fold-regulation of greater than two as up-regulated, and genes with a fold-regulation less than −2 as down-regulated. Compared to control cells, a total of six genes were significantly down-regulated following napabucasin treatment (*ALDH1A1*, *ATM*, *CD24*, *ID1*, *MUC1*, *PROM1*) and a total of seven genes were up-regulated (*DLL4*, *PLAUR*, *SIRT1*, *SNAI1*; significant for *JAG1*, *KLF4*, *LATS1*) (Figure 6). For a complete list of the results for all 84 genes measured, see Supplementary Table S2.

## 3. Discussion

Understanding the underlying mechanism of BTC development is essential for developing new and successful therapeutic strategies for this rare and fatal disease. Moreover, therapeutic substances that can be translated into clinical practice are of utmost importance. Napabucasin was described by Li et al. as a CSC inhibitor and is currently being used in numerous clinical studies [11] (clinicaltrials.gov). However, data regarding napabucasin and BTC are not yet available. Therefore, the aim of the present study was to evaluate napabucasin as a therapeutic substance in BTC, using a comprehensive in vitro model mirroring the cellular heterogeneity of BTC.

We found a general cytotoxic effect of napabucasin on all tested BTC cell lines, including cell lines that were more sensitive towards napabucasin (e.g., KKU-055), and cell lines that displayed a relatively high IC_50_ value after 72 h incubation (e.g., OCUG-1). For most cell lines, we found an IC_50_ around 1 µM, which matches existing publications regarding napabucasin in other cancer entities [12,15]. We further detected a concentration-dependent increase of apoptotic and necrotic cells following napabucasin treatment, which is in line with other studies. In osteosarcoma cells, treatment of cells with 1 µM napabucasin resulted in an increase of apoptotic and necrotic cells in a range similar to our measurements [15]. In human breast cancer cells, analogous results have been obtained, although these cells appear to be less sensitive to napabucasin [19]. In an in vivo study using a murine liver metastasis model by Guha et al., napabucasin treatment also led to induction of late apoptosis, further supporting the cytotoxic effect as caused by induction of apoptosis [20].

In the present study, the treatment of BTC cells with napabucasin diminished functional CSC traits, such as soft-agar colony formation and clonogenicity. Li and coworkers have described how napabucasin successfully inhibited the formation of tumor spheres in colorectal cancer cells [11]. Likewise, Zhang et al. wrote that napabucasin significantly diminished tumor sphere formation in prostate cancer stem cells [12]. In the current study, napabucasin significantly reduced the ability of BTC cells to form spheres to 10–80% of control samples, depending on the concentration. The effect of 0.60 µM napabucasin on sphere formation in NOZ cells was difficult to reproduce, meaning that in one experimental series we observed a clear negative effect on sphere formation, whereas in another series the effect was less prominent. When taking into account that 0.60 µM napabucasin only showed moderate general cytotoxicity, it is possible that a napabucasin concentration of 0.60 µM is at the border of affecting sphere formation and dependent on the well-being of the cells or their proliferative state, 0.60 µM has a more profound effect on sphere formation in one experiment than in another experiment. Interestingly, we found that the effect of napabucasin on sphere formation was also detectable when cells were pre-treated with napabucasin for 24 h without any further napabucasin treatment during the assay, suggesting a longer-lasting effect of napabucasin treatment on tumor sphere formation or an elimination of sphere-forming BTC cells due to the pre-treatment. Several publications demonstrated that the ALDH-positive cancer cell subpopulation harbors CSC traits [21,22]. In addition, there is evidence that ALDH-positive cells are also involved in BTC aggressiveness and represent part of the CSC phenotype in BTC [9,23]. In a work by MacDonagh and coworkers, the authors demonstrated that napabucasin decreases the number of ALDH-positive cells in cisplatin-resistant and non-resistant lung cancer cells [14]. Although we also found an effect of napabucasin on the ALDH-positive subpopulation in BTC cells, this effect was only evident for a relatively high napabucasin concentration and hard to reproduce, suggesting that lower concentrations did not affect ALDH-positivity in our experimental context. It should be noted that we found that napabucasin reduced CD326 surface expression in a non-concentration-dependent manner. This is interesting, since in our Western blot experiments, we measured a concentration-dependent reduction of this CSC marker.

The ability to form clones under conditions of very low seeding numbers is another widely-used approach to study CSC abilities. Our results show that even at low concentrations, napabucasin almost completely abolished clonogenic growth. Similar experiments with osteosarcoma cells and prostate cancer cells also demonstrated the negative effect of napabucasin on clonogenic growth [12,15]. In an interesting study by MacDonagh et al., the authors showed that combined treatment of napabucasin with cisplatin in cisplatin-resistant lung cancer cells significantly reduced clonogenic survival compared to chemotherapy alone, implying that napabucasin might be an effective adjuvant substance when used alongside standard chemotherapeutics [14].

In their initial study, Li et al. showed that napabucasin reduces protein expression of established CSC markers [11]. In accordance with these data, we also observed (a cell line-dependent) down-regulation of CSC markers. The tested cell lines showed a remarkable cell line-dependent composition regarding the chosen CSC panel, meaning that certain CSC markers were detectable in one cell line, but not in the other, and vice versa. This might be explainable simply by the fact that the cell lines are genetically different and from different origin, but also by the fact that different CSC subpopulations with different CSC marker composition may exist in BTC [6]. In this regard, it would be also interesting to isolate cells with (functional) CSC traits from BTC cells, and treat these cells with napabucasin to potentially identify CSC markers that make the cells more vulnerable or resistant towards napabucasin treatment.

The markers that were downregulated following napabucasin treatment in our study in BTC cells are (partially) different from the markers described by Li et al. [11]. This might be explainable by intrinsically different expression patterns of CSC markers, but also by varying effects of napabucasin in different tumor types. It is worth noting that, in agreement with the data presented by Li and coworkers, napabucasin also reduced the protein levels of CSC markers c-Myc and Nanog [11]. Interestingly, although we found concentration-dependent decline in Stat3 protein levels in both tested cell lines, pStat3 levels were not measurable (including control cells).

To cover a broader spectrum of CSC-related genes, we used the RT^2^ Profiler PCR Array comprising 84 human CSC-related genes (for a detailed list of the results of all 84 genes, see Supplementary Table S2). Treatment of BTC cells with 1.25 µM napabucasin for 24 h led to down-regulation of six genes and up-regulation of seven genes. While increased mRNA levels of CSC-related genes following napabucasin treatment is an unexpected result, a similar observation was published previously [14], when, similar to our results, napabucasin led to an increase of KLF4 expression, while in another study, napabucasin decreased KLF4 expression levels [12]. For KLF4, both a tumor-promoting as well as a tumor-suppressive role have been described [24,25]. For the other targets, the reasons for these contradictory results remain speculative and may encompass cellular compensation reactions towards napabucasin treatment or, like KLF4, tumor-specific multi-functionalities. 

In accordance with other studies, ALDH1 mRNA levels were significantly reduced following napabucasin treatment [11,14]. ALDH1 activity is linked to CSC properties, higher tumorigenic potential of BTC cells, and is known to be involved in early differentiation of stem cells [23]. Of note, as previously shown in BTC cells, increased ALDH1 activity led to higher expression levels of CSC CD24 and CD44 [9]. Furthermore, Shuang et al. found a relation between high ALDH1 expression levels and low overall survival in patients with intrahepatic and extrahepatic cholangiocarcinoma [23]. 

Treatment with napabucasin additionally resulted in a significant reduction of CD24 mRNA in our study, which is in line with results published by Li et al [11]. CD24 is an extracellular glycoprotein and cell membrane marker in CSC, and is known to increase tumor growth and promote metastasis [26]. Moreover, several studies reported that CD24 might be a CSC marker also for BTC [6]. PROM1 (also known as CD133) is another CSC surface marker linked to BTC that showed significant down-regulation following napabucasin treatment [6]. Combined with the observed down-regulation of CD24 after napabucasin treatment, the parallel downregulation of PROM1 could indicate that either mRNAs are downregulated independently, or that napabucasin may affect a certain CSC subpopulation expressing CD24 and PROM1 [7].

Previously, ATM has been detected to be down-regulated after napabucasin treatment in head and neck squamous carcinoma cells [11]. Likewise, we observed a significant reduction of ATM mRNA levels in BTC cells. Genetic alterations and overexpression of ATM have been described for several cancer types, including BTC [27,28,29]. Moreover, Nam et al. described ATM expression in HuCCt-1 cells [30]. Interestingly, in lung cancer stem-like cells, Chen and coworkers demonstrated that downregulation of ATM sensitizes cells towards radiotherapy, again suggesting that future studies might identify napabucasin as an adjuvant drug for conventional chemotherapeutics or radiotherapy in BTC and other cancer types.

MUC1 is a transmembrane protein that is overexpressed in several epithelial cancers, and is involved in various pro-oncogenic events, including CSC phenotype [31]. Interestingly, several studies have described high MUC1 expression in BTC [32,33], and napabucasin reduced MUC1 expression by about 3-fold.

ID1 is a basic helix-loop-helix transcription factor inhibiting transcription of tumor suppressors, such as p21 and p16 [34]. ID1 is deregulated in cancer, associated with poor prognosis, and contributes to stem cell phenotype [35,36,37]. In a study conducted by Harder et al., the authors measured ID1 expression in BTC patient samples. Although the authors could not demonstrate a significant correlation between ID1 expression and clinical prognosis, they observed deregulated expression of ID1 in BTC patients [38]. The current results in vitro indicate a 4-fold down-regulation of ID1 by napabucasin in BTC cells. 

In conclusion, using the RT^2^ Profiler Array, we detected that several CSC-related genes were expressed in BTC cells and that napabucasin caused significant down-regulation of several CSC-related genes. Future studies may investigate the role of these genes in BTC and elucidate their role in development and progression of BTC more in detail, especially with regard to clinical problems in BTC management, such as metastasis and tumor relapse.

In the present study, we first evaluated napabucasin as a potential compound in the treatment of BTC. We clearly demonstrated a cytotoxic effect of napabucasin in BTC cells, and a profound and significant anti-tumorigenic effect on functional CSC characteristics and CSC-relevant gene expression patterns. Our data might be the basis for subsequent, more detailed in vivo and additional in vitro studies regarding napabucasin and BTC, as well as for studies investigating the potential of napabucasin as an adjuvant substance combined with standard chemotherapeutics against BTC.

## 4. Materials and Methods

### 4.1. Cell Culture and Treatments

Nine cell lines established from biliary tract cancer (KKU-055, KKU-100, KKU-213), bile duct carcinoma (EGI-1, TFK-1, OZ, HuCCt-1 [39]), and gallbladder cancer (OCUG-1, NOZ) were used in this study. EGI-1 and TFK-1 were purchased from the German Collection of Microorganisms and Cell Cultures (DSZM; https://www.dsmz.de/), the other cells from the Japanese Collection of Research Bioresources Cell Bank (JCRB; http://cellbank.nibiohn.go.jp/english/). All cell lines were cultured under standard conditions at 37 °C in a humidified atmosphere containing 5% CO_2_ in high glucose Dulbecco’s modified Eagle’s medium (DMEM; Gibco, ThermoFisher Scientific, Vienna, Austria), supplemented with 10% (v/v) fetal bovine serum (FBS; Biochrom, Berlin, Germany), 1% antibiotic-antimycotic solution (ABAM, Sigma Aldrich, Darmstadt, Germany), 1 mM sodium pyruvate (Sigma Aldrich), and 10 mM HEPES (Sigma Aldrich).

Napabucasin (BBI608), was dissolved in 100% dimethyl sulfoxide (DMSO; Sigma Aldrich), according to the manufacturer’s protocol (Selleckchem, Houston, TX, USA), to a stock concentration of 20 mM. Aliquots were stored at −80 °C [11]. For control samples (UTC), an equivalent volume of the solvent DMSO was added.

For all experiments, cells (KKU-055, KKU-100, KKU-213, OZ, HuCCt-1, OCUG-1, NOZ) were seeded using 1.5 × 10^4^ cells per well in transparent 96-well plates (Starlab, Hamburg, Germany), and only EGi-1 and TFK-1 cells were seeded at a density of 2 × 10^4^ cells per well. If cells were seeded in 60 mm culture dishes (Starlab), 9.73 × 10^5^ (KKU-055, KKU-100, KKU-213, OZ, HuCCt-1, OCUG-1, NOZ) or 1.3 × 10^6^ (EGi-1, TFK-1) cells per dish were used. 

### 4.2. Napabucasin Cytotoxicity

For dose-dependent cytotoxicity of napabucasin, cells were seeded using the above-mentioned cell numbers in 96-well microplates, washed 24 h after seeding with serum-free medium (sfDMEM), and incubated with a 10-step napabucasin dilution series (0.04-20 µM) for 72 h. For incubation, we used sfDMEM to avoid potential interactions between napabucasin and serum components. However, since KKU-213 cells did significantly alter their morphology under serum-free conditions without napabucasin treatment, we used FBS-containing medium (10%) for this cell line. For each experiment, an untreated control with DMSO (UTC) was included. After 72 h incubation with napabucasin, viability was measured via resazurin (Sigma Aldrich) assay as described [40], on a Spark multimode reader (Tecan, Grödig, Austria), at an excitation wavelength of 535 nm, an emission wavelength of 588 nm, and optimal gain settings. The resazurin signal of treated samples was related to UTC cells. Each experimental series contained four technical replicates. Inhibitory concentration 50% values (concentration at which the viability of cells was reduced to 50%) were calculated for each cell line using linear interpolation.

Time-dependent cytotoxicity of napabucasin was measured using 0 (UTC), 0.6, 1.25, and 2.0 µM napabucasin after 0, 24, 48, and 72 h via the resazurin assay and the Spark multimode reader. Viability was related to the initial time points (0 h). Additionally, light microscopy images for each time point were made at the center of the 96-well microplate well with the imaging module of the Spark multimode reader.

### 4.3. Annexin V/7-AAD Staining

HuCCt-1 cells were seeded in 60 mm dishes, washed after 24 h once with serum-free media and incubated with 2.0, 1.25, or 0.6 µM napabucasin for additional 24 h. Untreated control samples were included in each experimental series. The Annexin V assay was performed following the manufacturer’s protocol (Biolegend, San Diego, CA, USA), and analyzed by flow cytometry (Cell Lab Quanta SC, Beckman Coulter, Brea, CA, USA) using the Kaluza Analysis 1.3 software (Beckman Coulter).

### 4.4. Soft Agar Assay

To monitor anchorage-independent growth, the soft agar colony formation assay (CytoSelect 96-well Cell Transformation Assay; Cell Biolabs, San Diego, CA, USA) was used for HuCCt-1 and NOZ cells according to the manufacturers’ instructions. For pre-incubation experiments, cells were seeded in 25 cm^2^ flasks, grown overnight and incubated with 0.6, 1.25, or 2.0 µM napabucasin for the subsequent 24 h. Cells were harvested and 2 × 10^5^ cells per ml were seeded in a 96-well plate containing agar solution in high glucose Dulbecco’s modified Eagle’s medium (DMEM; Gibco, ThermoFisher Scientific, Vienna, Austria), supplemented with 10% (v/v) fetal bovine serum (FBS; Biochrom), 1% antibiotic-antimycotic solution (ABAM, Sigma Aldrich), 1 mM sodium pyruvate (Sigma Aldrich) and 10 mM HEPES (Sigma Aldrich,), and without napabucasin, grown for eigth days and analyzed for sphere formation. For non-pre-incubation experiments, 2 × 10^5^ cells per ml were seeded in a 96-well plate containing agar solution in DMEM (10% FBS), containing 0.6, 1.25 or 2.0 µM napabucasin, respectively. Control cells (DMSO-only) were included in each experimental series. After eight days, sphere formation was quantified with the CyQuant GR Dye (Cell Biolabs) after cell lysis, and measured with the Spark multimode reader at 485 nm excitation and 520 nm emission wavelengths. Visual analysis of each condition was made after eight days with a CKX53 inverted microscope equipped with a monochrome XM10 digital camera (Olympus, Vienna, Austria).

### 4.5. Clonogenic Assay

HuCCt-1 and NOZ cells were seeded in serum-containing media in a 96-well microplate in low concentrations: 60 cells per well for HuCCt-1 and 30 cells per well for NOZ cells. Cells were allowed to attach overnight, carefully washed with media, and incubated with 0.6, 1.25, and 2.0 µM napabucasin. For these experiments, incubation was performed using serum-containing media, since at these low seeding numbers, cells were not able to survive in sfDMEM. Plates were incubated for up to 6 days in a humidified atmosphere containing 5% CO_2_ and at 37 °C. The confluence measurement function of the Spark multimode reader (Tecan) was used to monitor clonogenic growth (non-endpoint), as described before [18]. Clonogenic growth was measured after 3, 4, 5, and 6 days. ImageJ (V1.48, NIH, Bethesda, MD, USA) was used for counting and measurement of colony mean size [18,41]. Untreated control cells were included in each experimental series.

### 4.6. Aldehyde Dehydrogenase 1 Assay and CD326 Expression

Since NOZ cells were not suitable for flow cytometer analysis, only HuCCt-1 were used for these experiments. The ALDEFLUOR Kit (Stemcell Technologies, Grenoble, France) was used to identify and quantify ALDH-positive subpopulations in HuCCt-1 cells, and a specific CD326 antibody (Miltenyi Biotec, Bergisch Gladbach, Germany) was used for detection of CD326 (EpCAM, epithelial cell adhesion molecule). Following the manufacturer’s protocol, cells were seeded in 60 mm dishes, grown overnight and treated with 0.6, 1.25, or 2.0 µM napabucasin for 24 h. Cells were stained with Aldefluor reagent or CD326 antibody for 30 minutes according to the manufacturer’s instructions, and analyzed with a Cell Lab Quanta SC flow cytometer and the Kaluza Analysis 1.3 software (Beckman Coulter). Untreated controls were included in each experimental series. 

### 4.7. Western Blot

For Western blot analysis, cells (HuCCt-1 and NOZ) were seeded in 60 mm dishes, grown overnight and incubated for 24 h with 0.6, 1.25, or 2.0 µM napabucasin or DMSO (controls). Cells were harvested, centrifuged, resuspended in phosphate-buffered saline (PBS), counted, and frozen as pellets at −20 °C until subsequent processing. Frozen cell pellets were resuspended in a defined volume of PBS to obtain a cell concentration of 1 × 10^7^ cells per ml. Cells were lysed by sonification (Sonopuls HD70, Bandelin, Berlin, Germany) and centrifuged. Ten µl of the supernatant (containing material from 10^5^ cells) were mixed with 10 µl 2× sodium dodecyl sulfate (SDS) sample buffer and incubated for 5 min at 95 °C. Samples were loaded on SDS gels (4-20% Mini-PROTEAN TGX, BioRad, Vienna, Austria) and let run for 70 minutes at 100 V. For Western blotting, the Trans-Blot Turbo Mini Nitrocellulose Transfer Packs System (BioRad) was used. Blots were incubated overnight at 4 °C with primary antibodies at defined concentrations (see Supplementary Table S1). After three washing steps, blots were incubated at room temperature for 1 h with the secondary antibody. Signals were developed using the Signal Fire ECL Reagent (Cell Signaling Technology, Danvers, MA, USA) and protein bands were detected using a ChemiDoc MP System (BioRad). Measurement of gray densities of the bands with ImageJ was used for quantification.

### 4.8. Gene Expression Analysis

Total RNA was isolated from HuCCt-1 cells with a RNeasy Mini Kit and QIAshredder tubes (Qiagen, Hilden, Germany) according to the manufacturer’s protocol. Cells were seeded in 60 mm dishes, grown overnight, and incubated for 24 h with 1.25 µM napabucasin and an untreated control only containing DMSO. cDNA synthesis was performed with the RT^2^ First Strand Kit (Qiagen) according to the manufacturer’s instructions. 1 µg of total RNA (quantified by photometry, BioPhotometer, Eppendorf, Hamburg, Germany) was used for reverse transcription. For quantitative real-time PCR, a Qiagen RT^2^ Profiler PCR Array Human Cancer Stem Cells for a 384-well plate was used. Gene expression was measured on a ViiA7 real-time PCR System (Applied Biosystems, ThermoFisher Scientific, Vienna, Austria). For data analysis and sample normalization, Qiagen’s Data Analysis Center was used and samples were related to five housekeeping genes (*β-actin*, *b2m*, *gapdh*, *hprt1*, *rplp0*) included on the plate according to the manufacturer’s protocol. Up-regulated genes (fold change greater than two) are represented as ΔΔCt values related to untreated controls, down-regulated genes (fold change less than two) are represented as −(1/fold change) related to untreated controls, according to the Qiagen Data Analysis Center.

### 4.9. Statistics

All data points represent mean values of at least three biological replicates (*n* ≥ 3) ± standard error of mean (SEM). A paired student’s *t*-test was used after analysis of normal distribution for calculation of significances between control and treated samples. All calculations were performed using OriginPro 9.1 (OriginLab, Northampton, MA, USA). Statistical results were considered significant (*) or highly significant (**) at *p* < 0.05 and *p* < 0.01, respectively.

## Supplementary Materials

The following are available online at https://www.mdpi.com/2072-6694/11/3/276/s1, Supplementary Figure S1, representative images of time-resolved cytotoxic effect of napabucasin in biliary tract cancer cell lines. Table S1, list of antibodies used for protein expression analysis. Table S2. Supplementary data for Figure 5, complete gene list of cancer stem cell-related genes measured with the RT^2^ Profiler PCR Array.

## References

[1] Razumilava N., Gores G.J. (2014). Cholangiocarcinoma. Lancet.

[2] Valle J.W., Furuse J., Jitlal M., Beare S., Mizuno N., Wasan H., Bridgewater J., Okusaka T. (2014). Cisplatin and gemcitabine for advanced biliary tract cancer: A meta-analysis of two randomised trials. Ann. Oncol..

[3] Kreso A., Dick J.E. (2014). Evolution of the cancer stem cell model. Cell Stem Cell.

[4] Oikawa T. (2016). Cancer Stem cells and their cellular origins in primary liver and biliary tract cancers. Hepatology.

[5] Franco S.S., Szczesna K., Iliou M.S., Al-Qahtani M., Mobasheri A., Kobolak J., Dinnyes A. (2016). In vitro models of cancer stem cells and clinical applications. BMC Cancer.

[6] Mayr C., Ocker M., Ritter M., Pichler M., Neureiter D., Kiesslich T. (2017). Biliary tract cancer stem cells–translational options and challenges. World J. Gastroenterol..

[7] Kemmerling R., Alinger B., Dietze O., Bosmuller H.C., Ocker M., Wolkersdorfer G.W., Berr F., Neureiter D., Kiesslich T. (2012). Association of stem cell marker expression pattern and survival in human biliary tract cancer. Int. J. Oncol..

[8] Bekaii-Saab T., El-Rayes B. (2017). Identifying and targeting cancer stem cells in the treatment of gastric cancer. Cancer.

[9] Mayr C., Wagner A., Stoecklinger A., Jakab M., Illig R., Berr F., Pichler M., Di Fazio P., Ocker M., Neureiter D. (2015). 3-Deazaneplanocin A May Directly Target Putative Cancer Stem Cells in Biliary Tract Cancer. Anticancer Res..

[10] Mayr C., Wagner A., Loeffelberger M., Bruckner D., Jakab M., Berr F., Di Fazio P., Ocker M., Neureiter D., Pichler M. (2016). The BMI1 inhibitor PTC-209 is a potential compound to halt cellular growth in biliary tract cancer cells. Oncotarget.

[11] Li Y., Rogoff H.A., Keates S., Gao Y., Murikipudi S., Mikule K., Leggett D., Li W., Pardee A.B., Li C.J. (2015). Suppression of cancer relapse and metastasis by inhibiting cancer stemness. Proc. Natl. Acad. Sci. USA.

[12] Zhang Y., Jin Z., Zhou H., Ou X., Xu Y., Li H., Liu C., Li B. (2016). Suppression of prostate cancer progression by cancer cell stemness inhibitor napabucasin. Cancer Med..

[13] Hubbard J.M., Grothey A. (2017). Napabucasin: An Update on the First-in-Class Cancer Stemness Inhibitor. Drugs.

[14] MacDonagh L., Gray S.G., Breen E., Cuffe S., Finn S.P., O’Byrne K.J., Barr M.P. (2018). BBI608 inhibits cancer stemness and reverses cisplatin resistance in NSCLC. Cancer Lett..

[15] Zuo D., Shogren K.L., Zang J., Jewison D.E., Waletzki B.E., Miller A.L., Okuno S.H., Cai Z., Yaszemski M.J., Maran A. (2018). Inhibition of STAT3 blocks protein synthesis and tumor metastasis in osteosarcoma cells. J. Exp. Clin. Cancer Res..

[16] Charafe-Jauffret E., Ginestier C., Birnbaum D. (2009). Breast cancer stem cells: tools and models to rely on. BMC Cancer.

[17] Buick R.N., MacKillop W.J. (1981). Measurement of self-renewal in culture of clonogenic cells from human ovarian carcinoma. Br. J. Cancer.

[18] Mayr C., Beyreis M., Dobias H., Gaisberger M., Pichler M., Ritter M., Jakab M., Neureiter D., Kiesslich T. (2018). Miniaturization of the Clonogenic Assay Using Confluence Measurement. Int. J. Mol. Sci..

[19] Locken H., Clamor C., Muller K. (2018). Napabucasin and Related Heterocycle-Fused Naphthoquinones as STAT3 Inhibitors with Antiproliferative Activity against Cancer Cells. J. Nat. Prod..

[20] Guha P., Gardell J., Darpolor J., Cunetta M., Lima M., Miller G., Espat N.J., Junghans R.P., Katz S.C. (2018). STAT3 inhibition induces Bax-dependent apoptosis in liver tumor myeloid-derived suppressor cells. Oncogene.

[21] Douville J., Beaulieu R., Balicki D. (2009). ALDH1 as a functional marker of cancer stem and progenitor cells. Stem Cells Dev..

[22] Charafe-Jauffret E., Ginestier C., Bertucci F., Cabaud O., Wicinski J., Finetti P., Josselin E., Adelaide J., Nguyen T.T., Monville F. (2013). ALDH1-positive cancer stem cells predict engraftment of primary breast tumors and are governed by a common stem cell program. Cancer Res..

[23] Shuang Z.Y., Wu W.C., Xu J., Lin G., Liu Y.C., Lao X.M., Zheng L., Li S. (2014). Transforming growth factor-beta1-induced epithelial-mesenchymal transition generates ALDH-positive cells with stem cell properties in cholangiocarcinoma. Cancer Lett..

[24] Muller M., Hermann P.C., Liebau S., Weidgang C., Seufferlein T., Kleger A., Perkhofer L. (2016). The role of pluripotency factors to drive stemness in gastrointestinal cancer. Stem Cell Res..

[25] Yu M., Hao B., Zhan Y., Luo G. (2018). Kruppel-like factor 4 expression in solid tumor prognosis: A meta-analysis. Clin. Chim. Acta.

[26] Saeg F., Anbalagan M. (2018). Breast cancer stem cells and the challenges of eradication: A review of novel therapies. Stem Cell Investig..

[27] Xu Y., Gao P., Lv X., Zhang L., Zhang J. (2017). The role of the ataxia telangiectasia mutated gene in lung cancer: recent advances in research. Ther. Adv. Respir. Dis..

[28] Angele S., Falconer A., Foster C.S., Taniere P., Eeles R.A., Hall J. (2004). ATM protein overexpression in prostate tumors: possible role in telomere maintenance. Am. J. Clin. Pathol..

[29] Morizane C., Ueno M., Ikeda M., Okusaka T., Ishii H., Furuse J. (2018). New developments in systemic therapy for advanced biliary tract cancer. Jpn J. Clin. Oncol..

[30] Nam A.R., Jin M.H., Park J.E., Bang J.H., Oh D.Y., Bang Y.J. (2018). Therapeutic Targeting of the DNA Damage Response Using an ATR Inhibitor in Biliary Tract Cancer. Cancer Res. Treat..

[31] Nath S., Mukherjee P. (2014). MUC1: A multifaceted oncoprotein with a key role in cancer progression. Trends Mol. Med..

[32] Wan X., Shi J., Wang A., Xie Y., Yang X., Zhu C., Zhang H., Wu L., Wang S., Huang H. (2017). Gallbladder papillary neoplasms share pathological features with intraductal papillary neoplasm of the bile duct. Oncotarget.

[33] Ruys A.T., Groot Koerkamp B., Wiggers J.K., Klumpen H.J., ten Kate F.J., van Gulik T.M. (2014). Prognostic biomarkers in patients with resected cholangiocarcinoma: a systematic review and meta-analysis. Ann. Surg. Oncol..

[34] Tang J., Gordon G.M., Nickoloff B.J., Foreman K.E. (2002). The helix-loop-helix protein ID-1 delays onset of replicative senescence in human endothelial cells. Lab. Invest..

[35] Perk J., Iavarone A., Benezra R. (2005). Id family of helix-loop-helix proteins in cancer. Nat. Rev. Cancer.

[36] Hasskarl J., Munger K. (2002). Id proteins--tumor markers or oncogenes?. Cancer Biol. Ther..

[37] Ying Q.L., Nichols J., Chambers I., Smith A. (2003). BMP induction of Id proteins suppresses differentiation and sustains embryonic stem cell self-renewal in collaboration with STAT3. Cell.

[38] Harder J., Muller M.J., Fuchs M., Gumpp V., Schmitt-Graeff A., Fischer R., Frank M., Opitz O., Hasskarl J. (2013). Inhibitor of differentiation proteins do not influence prognosis of biliary tract cancer. World J. Gastroenterol..

[39] Miyagiwa M., Ichida T., Tokiwa T., Sato J., Sasaki H. (1989). A new human cholangiocellular carcinoma cell line (HuCC-T1) producing carbohydrate antigen 19/9 in serum-free medium. In Vitro Cell Dev. Biol..

[40] Kiesslich T., Neureiter D., Alinger B., Jansky G.L., Berlanda J., Mkrtchyan V., Ocker M., Plaetzer K., Berr F. (2010). Uptake and phototoxicity of meso-tetrahydroxyphenyl chlorine are highly variable in human biliary tract cancer cell lines and correlate with markers of differentiation and proliferation. Photochem. Photobiol. Sci..

[41] Rafehi H., Orlowski C., Georgiadis G.T., Ververis K., El-Osta A., Karagiannis T.C. (2011). Clonogenic assay: Adherent cells. J. Vis. Exp..

