# Supplementary material for: The Cancer Stem Cell Inhibitor Napabucasin (BBI608) Shows General Cytotoxicity in Biliary Tract Cancer Cells and Reduces Cancer Stem Cell Characteristics
Source: Cancers (Basel). 2019 Feb 26;11(3):276. doi: 10.3390/cancers11030276 (PMC6468451; doi:10.3390/cancers11030276)
Supplement: Supplementary file 1 [file cancers-11-00276-s001.zip › cancers-443450-supplementary/Figure S1.pdf]

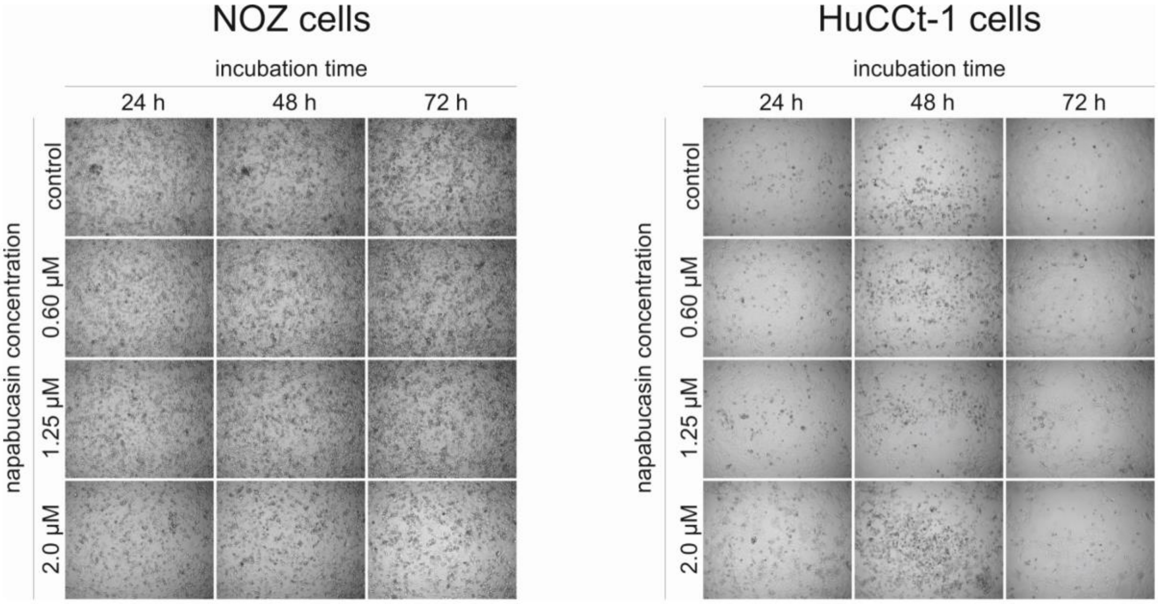

Figure S1. Representative images of time-resolved cytotoxic effect of napabucasin in biliary tract cancer cell lines
